# Comparison between Chest-Worn Accelerometer and Gyroscope Performance for Heart Rate and Respiratory Rate Monitoring

**DOI:** 10.3390/bios12100834

**Published:** 2022-10-06

**Authors:** Chiara Romano, Emiliano Schena, Domenico Formica, Carlo Massaroni

**Affiliations:** 1 Unit of Measurements and Biomedical Instrumentation, Department of Engineering, Università Campus Bio-Medico di Roma, Via Alvaro del Portillo, 21, 00128 Rome, Italy; 2 School of Engineering, Newcastle University, Newcastle upon Tyne NE1 7RU, UK

**Keywords:** heart rate, respiratory rate, wearable systems, mechanical vibrations, magneto-inertial measurement units

## Abstract

The demand for wearable devices to simultaneously monitor heart rate (HR) and respiratory rate (RR) values has grown due to the incidence increase in cardiovascular and respiratory diseases. The use of inertial measurement unit (IMU) sensors, embedding both accelerometers and gyroscopes, may ensure a non-intrusive and low-cost monitoring. While both accelerometers and gyroscopes have been assessed independently for both HR and RR monitoring, there lacks a comprehensive comparison between them when used simultaneously. In this study, we used both accelerometers and gyroscopes embedded in a single IMU sensor for the simultaneous monitoring of HR and RR. The following main findings emerged: (i) the accelerometer outperformed the gyroscope in terms of accuracy in both HR and RR estimation; (ii) the window length used to estimate HR and RR values influences the accuracy; and (iii) increasing the length over 25 s does not provide a relevant improvement, but accuracy improves when the subject is seated or lying down, and deteriorates in the standing posture. Our study provides a comprehensive comparison between two promising systems, highlighting their potentiality for real-time cardiorespiratory monitoring. Furthermore, we give new insights into the influence of window length and posture on the systems’ performance, which can be useful to spread this approach in clinical settings.

## 1. Introduction

Taking care of oneself and paying attention to one’s state of health are topics that are registering a rising interest among the population. Indeed, over the past few years, there has been an increasing willingness to continuously monitor health status with the aim of reducing risk by means of early diagnosis, healthy lifestyle, and prevention. The reason lies in the strong correlation between vital parameters and a variety of physiological, psychological, and environmental stressors [1,2,3]. Monitoring vital parameters on an ongoing basis, especially heart rate (HR) and respiratory rate (RR) values, can be useful in several fields of applications, ranging from occupational to sports environment due to their sensitivity to cognitive load, stress, and other factors [4,5].

Nowadays, the ECG is the most widely used technique for the diagnosis of heart-related diseases; however, for years, research has been interested in alternative methods, e.g., based on photoplethysmography (PPG) and ballistocardiography (BCG) [6,7]. Nevertheless, RR is typically assessed by devices based on strain sensors and impedance plethysmography [8], or alternatively from the modulation of an ECG waveform caused by breathing [9]. Among all wearable devices, interest in IMU sensors has exploded in recent years, mainly due to the miniaturization of electronic components, extended battery life, and improved data management. These features have made them an attractive choice for continuous non-invasive monitoring with low cost, low power consumption, and privacy-friendly characteristics [10,11].

**Figure 1:**
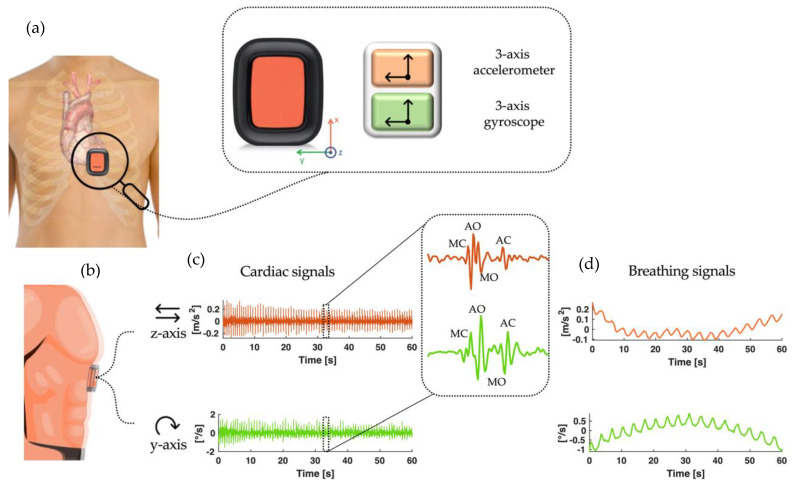
(**a**) Schematic illustration of the positioning of the IMU sensor (embedding the ACC and the GYR) on the subject’s chest from the frontal view. (**b**) Lateral view of the IMU sensor on the subject’s chest. Note that the sensor is integral with the body of the subject. This ensures that acceleration and angular velocity measurements correspond with good approximation to those experienced by the chest in response to cardiac and respiratory activity. (**c**) Example of an acceleration (in orange) and angular velocity (in green) signal filtered so that only the cardiac component is reported. (**d**) Example of an acceleration (in orange) and angular velocity (in green) signal filtered so that only the respiratory component is reported.

The output of IMU sensors embedding both an accelerometer (ACC) and a gyroscope (GYR) may allow the simultaneous monitoring of both cardiac and respiratory mechanics, also providing additional information to conventional techniques used to monitor these vital parameters [6,12,13]. The cardiac activity can be assessed through the seismocardiogram (SCG) and the gyrocardiogram (GCG) signals by measuring local vibrations (in terms of accelerations and angular velocities) in response to heart ejection [6]. The SCG is defined as the study of body vibrations induced by the heartbeat and was popularized in the 1990s by Salerno and Zanetti [14,15]. Only recently, it has been augmented by the GCG, which locally measures the angular velocities produced at the precordial level by the cardiac activity [13,16]. Respiratory activity can be extracted by breathing-induced movements of the rib cage impressed in the sensors’ output. Thus, the acceleration and angular velocity along one of the three axes will present both waveforms characteristic of cardiac vibrations and breathing movements, as shown in Figure 1. HR and RR estimation from the ACC and GYR integrated in IMU sensors has been already proposed in the literature with promising results [12,13]. Although the ACC is the most popular, the literature suggests that up to 60% of cardiac vibrational energy is contained in the gyration signal [17], indicating that the angular velocity signal has a higher noise rejection ratio than the acceleration signal [16,18]. For these reasons, the GYR output is widely used as an enhancement of the ACC signal. In [19], a method to improve the fusion of an ACC and GYR sensor by using a Kalman filter is suggested. In [20], authors propose real-time cardiac beat detection and heart rate monitoring using a combination of ACC and GYR output signals to improve accuracy due to fundamentally different noise rejection criteria. In [21], authors describe a home health monitoring solution with cardiac beat-to-beat detection using ACC and GYR signal fusion. Finally, in [22], an enhanced method to estimate HR values by combining the six-axis ACC and GYR signals is used. It is worth noting that only a few studies investigated the simultaneous measurement of HR and RR values, and they performed the estimation using only the ACC sensor. In addition, these analyses were rarely carried out in different postures of the subject [23,24].

Hence, while both ACCs and GYRs have been used to estimate HR or RR values, to the best of our knowledge, neither provide differences in terms of performance between these two sensors in the simultaneous extraction of HR and RR values.

In this study, we provided a comparison between ACCs and GYRs used for the simultaneous extraction of HR and RR values by using a frequency domain analysis. Since the signals are sensitive to physical conditions and postures, the subjects were experimented in three different postures (i.e., sitting, lying down, and standing). Given the variability of the heart and respiratory rate ranges within each trial, the identification of HR and RR values has been performed by considering a moving time window with a sliding step of 1 s. We carried out the analysis in moving windows of different lengths with the purpose of investigating how this parameter influences the assessment of HR and RR values.

The main contributions of this study can be summarized as follows: (1) to provide a comparison between the performances of ACCs and GYRs in the simultaneous extraction of HR and RR values; (2) to investigate how the window length influences the estimation of HR and RR values; and (3) to test the system in various measurement conditions (i.e., the subjects’ posture) for a more practical application.

## 2. Materials and Methods

### 2.1. Cardiorespiratory Monitoring by IMU Sensors

The IMU sensors typically embed a tri-axis ACC and a tri-axis GYR and are generally manufactured by microelectromechanical system (MEMS) technology.

If the IMU sensor is placed integral to the subject’s chest, it can measure the accelerations and angular velocities of the chest itself induced by cardiac and respiratory activity along three axes: the dorso–ventral axis (back-to-front), the superior–inferior axis (head-to-foot), and the sinistro–dexter axis (left-to-right) [6,25]. The breathing activity generates in the acceleration and angular velocity signals a slow-varying pattern that reflects the expansion and contraction of the chest caused by the movement of air in the lungs and the simultaneous movement of the upper chest. On the other hand, the heartbeat generates compression waves spreading throughout the thorax that produce vibrations in the sternum. The recording of minute body accelerations and angular velocities induced by cardiac activity at the precordial level can be collected as SCG and GCG signals, respectively. These signals contain peaks that reflect physiological events in the heart, such as mitral valve closure (MC) and aortic valve opening (AO) peaks, related to systolic activity, and aortic valve closure (AC) and mitral valve opening (MO) peaks, related to diastolic motion (see Figure 1). The AO peak is the most prominent and has been widely used in many studies for HR estimation [12,26].

### 2.2. Study Design

Eleven healthy volunteers were enrolled in this study. To each volunteer, a single IMU sensor (Xsens DOT, by Xsens), embedding a triaxial ACC (full scale ± 16 g) and a triaxial GYR (full scale ± 2000°/s), was fixed at the fifth-left intercostal space in the midclavicular line of the mitral valve [27]. The small size (36 × 30 × 11 mm) and mass (11.2 g) of this device gave the sensor optimal adherence to the subject’s chest without introducing discomfort to the user and ensuring a good coupling effect between the sensor and the body. This ensures that the two systems (i.e., the body and the sensor) are integral. Data were collected at 120 Hz and saved in the internal memory of the device.

The wearable chest multiparametric device Zephyr Bioharness ™ (Zephyr Technology Corporation, Annapolis, MD, USA) was used as a reference system for recording both the ECG waveform (at 250 Hz sampling rate) and the respiratory waveform (at 25 Hz sampling rate) [28]. The Bioharness system is a U.S. FDA-approved physiological monitoring device that consists of a chest strap and an electronic module. It acquires the ECG waveform via dry electrodes and the user’s breathing pattern through an embedded proprietary capacitive sensor. Based upon the principle of a strain gauge sensor (i.e., the resistance of a conductor is increased when the area of the conductor is increased), thoracic expansion and contraction cause size differentials that induce changes in capacitance because of resultant changes in the impedance. The change in impedance is manifested as a change in the waveform signal amplitude, represented as a sine wave with downward and upward deflections, indicating chest expansion (increased impedance) and contraction (decreased impedance), respectively.

**Figure 2:**
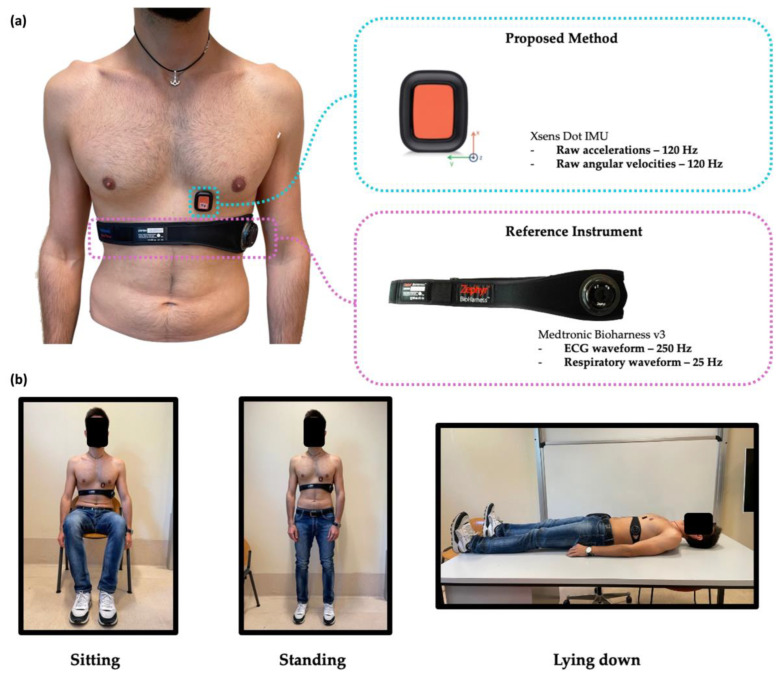
(**a**) Positioning of the IMU sensor at the fifth-left intercostal space in the midclavicular line of the mitral valve and of the wearable chest multiparametric device used as a reference system for registering both the ECG waveform and the respiratory waveform. (**b**) Picture of one subject during the protocol performed in the 3 at-rest postures, i.e., sitting, standing, and lying down.

Each volunteer was asked to carry out a protocol that sequentially included three different at-rest postures: sitting for ~2 min, standing for ~2 min, and lying down for ~2 min, as schematically reported in Figure 2. In each posture, the subject was asked to breathe quietly and hold their breath at the end for 20 s. The study was conducted in accordance with the Declaration of Helsinki and the study design was approved by the Ethical Committee of University Campus Bio-Medico di Roma (code: 27.2(18).20 of 15 June 2020).

### 2.3. Signal Pre-Processing

As briefly described in Section 2.1, the raw acceleration and angular velocity signals were modulated by both the cardiac and respiratory activity components. Therefore, the data were collected and pre-processed separately for the two activities to emphasize the features of each one.

#### 2.3.1. Cardiac Activity

Raw data were collected along 3 axes (i.e., x, y, and z, respectively). Although there was information content in each axis, we chose to select a single axis for each signal to reduce the problem’s dimensionality. Specifically, the *z* axis and the *y* axis were selected for the ACC and GYR output signals, respectively, in line with the literature that points to them as the most promising for this type of analysis [6,21,29,30]. Afterward, the signal was reconstructed by using the inverse continuous wavelet transform (icwt) between 10 Hz and 40 Hz in order to eliminate frequencies associated with slow-varying trends (e.g., respiratory activity) and isolate only the high-frequency packets representative of cardiac activity. The choice to use icwt was made because it allows for greater selectivity in the frequency band of interest, compared with classical bandpass filters, allowing noise cancellation to be performed without distortion of the raw signal [23,31,32,33]. Then, the root mean square envelope with a sliding window of 40 samples was applied to the reconstructed signal to emphasize each heartbeat.

Thus, the signal calculated described above has a different shape from the original signal and takes on a shape, almost like a periodic wave. Finally, a 1st order Butterworth band-pass filter between 0.7 Hz and 3 Hz was applied on the whole signal (i.e., 120 s of acquisition for each subject and each posture). The choice of these cutoff frequencies (i.e., 0.7 Hz and 3 Hz) was chosen according to the frequency components of vibrations induced by the blood flow ejection into the vascular bed and allowed the presence of the potential source of noise, such as motion artifacts, to be attenuated.

**Figure 3:**
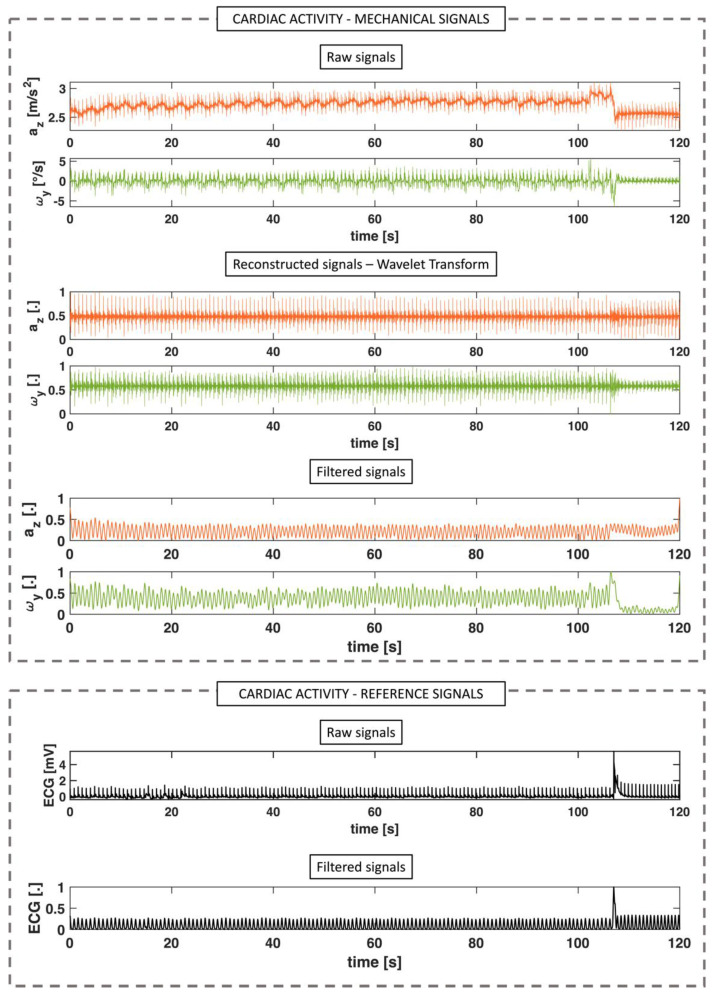
Signal processing for cardiac activity extraction for mechanical signals (i.e., accelerations, *a* and angular velocities, ω) and reference signal (ECG). After acquiring the raw signals of the ACC and the GYR, only one axis was selected to reduce the dimensionality of the problem (i.e., the *z* axis for the ACC and the *y* axis for the GYR). The selected signals were reconstructed using the inverse wavelet transform by selecting frequencies between 10 Hz and 40 Hz and normalized. Finally, the normalized signal envelope was extracted to emphasize the heartbeats and filtered between 0.7 and 3 Hz. Concurrently, the raw ECG reference signal was filtered between 0.7 and 3 Hz.

Concurrently, we applied a 1st order Butterworth bandpass filter between 0.7 Hz and 3 Hz on the recorded reference ECG waveform, to emphasize only the R wave of the QRS complex. An example of the signal processing for the cardiac activity analysis is shown in Figure 3.

#### 2.3.2. Respiratory Activity

**Figure 4:**
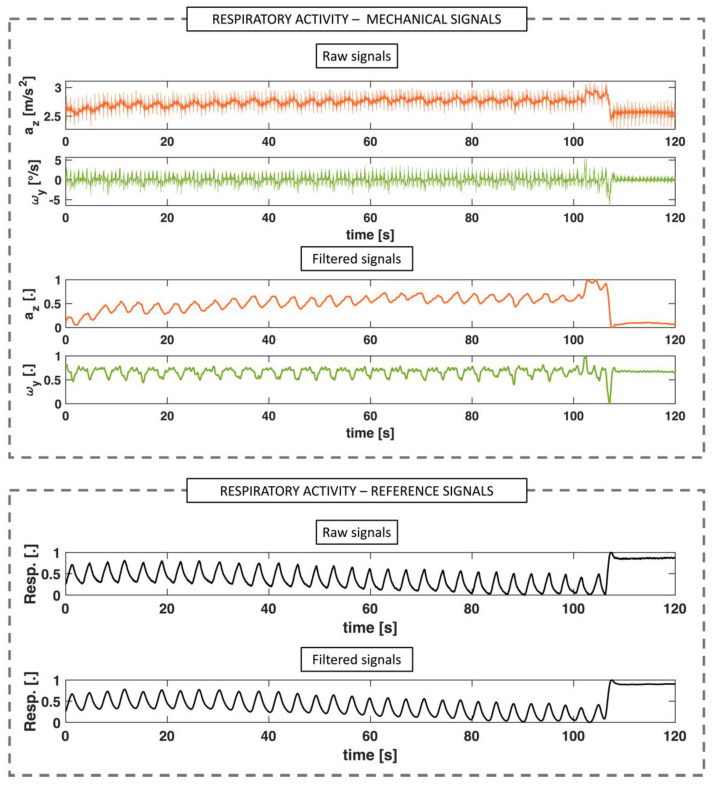
Signal processing for respiratory activity extraction for mechanical signals (i.e., accelerations, *a* and angular velocities, *ω*) and reference respiratory waveform.

Only one axis was also selected from each sensor (i.e., the *z* axis for the ACC and the *y* axis for the GYR) to monitor the respiratory activity [34]. For the RR assessment using the ACC and the GYR, the reference respiratory signals were pre-filtered with a Butterworth band-pass filter with cut-off frequencies of 0.1 Hz and 0.7 Hz to avoid the slow signal variations unrelated to respiratory activity and to eliminate high frequencies due to both noise and cardiac activity. A bandpass configuration was chosen, by fixing the low cut-off frequency around 0.1 Hz, to avoid the slow signal variations unrelated to respiratory movements and a high cut-off frequency around 0.7 Hz. In this way, the changes generated by the respiratory movements recorded can be adequately isolated and relayed to the subsequent elaboration stages. An example of signal processing for the analysis of respiratory activity is shown in Figure 4.

#### 2.3.3. Signal Windowing for HR and RR Extraction

After the signal pre-processing, we extracted the HR and RR values using a frequency domain analysis. However, using the power spectral density (PSD) on the whole signal did not allow us to have any information on the variations in HR and RR values over time, but it provides only a mean value. Hence, after the pre-processing stage, the filtered signals were segmented by using moving windows with a sliding step of 1 s. In each window, we selected the dominant frequency of the signal, obtaining the HR and RR variations over time with an update time of 1 s and accuracy (in terms of temporal variations detected) related to the length of the signal’s portion analyzed in each window, which is thus related to the length of the window used.

The same frequency extraction was performed by using six different sliding windows with sizes ranging from 5 s to 55 s in steps of 10 s.

The frequency range was chosen from 5 s to 55 s to include extreme cases and to find out the right trade-off between temporal resolutions of HR and RR values provided (related to the window size) and system performance. In fact, when a 5 s window was used, the selected signal did not include an entire respiratory act under physiological conditions (in eupnea, the subject could breathe at a rate lower than 12 BrPM). In this case, the technique based on FFT cannot provide a reliable RR estimation. Conversely, the use of a 55 s window implies averaging over a larger portion of the signal with a consequent minimization of errors caused by noise. However, quick changes in the phenomenon were averaged and masked.

Therefore, firstly, both mechanical and reference pre-filtered signals were segmented with different window sizes. Secondly, the PSD was applied to the signal segments using the Welch’s method with a window length equal to the length of the portion of the previously segmented signal. The PSD was calculated by applying a 0% overlap and with zero padding. The analysis in the spectral domain was carried out to estimate the values of HR and RR both from the reference system and from the mechanical signals (i.e., the ACC and the GYR). Therefore, the reference system was firstly used to obtain the ECG and respiratory waveforms. Then, these signals were band-pass-filtered in order to attenuate frequencies that were not related to cardiac and respiratory activities and to make signals more periodic by emphasizing beats (in the case of the ECG) and the inspiratory peaks in the case of the respiratory waveform. Finally, HR and RR values were extracted with a frequency domain analysis in accordance with [35,36] where a similar analysis was used to extract HR and RR reference values from the Bioharness. Thus, of each signal, the PSD whose maximum peak corresponds to the frequency with the highest power in the signal was evaluated. The latter was selected and multiplied by 60 s·min^−1^ in order to calculate the HR and RR expressed in bpm and BrPM, respectively.

It is noteworthy that the PSD resolution depends on the number of samples in the window itself. Specifically, the spectral resolution increased when longer time windows were considered, with a concomitant reduction in real-time performances (longer time windows require a longer wait for a result). This means that different window sizes would have different resolutions and the results would be hardly comparable. For this reason, a constant resolution of 0. 166 mHz (i.e., 0.01 bpm for the HR and 0.01 BrPM for the RR) was set by setting the ratio between the sampling rate of the signal and the number of discrete Fourier transform points to use in the PSD estimate. With this aim, the data related to both cardiac activity and respiratory activity were zero-padded.

**Figure 5:**
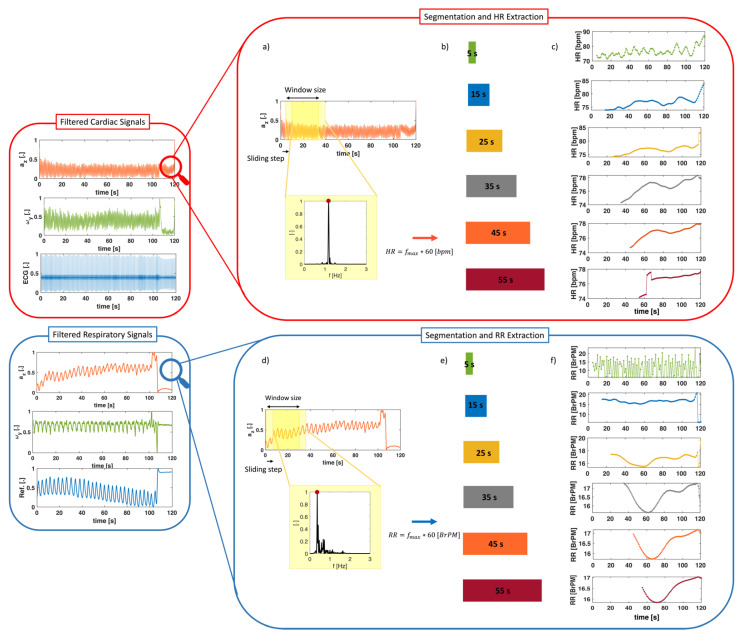
(**a**,**d**) After signal preprocessing, HR and RR values were extracted from both mechanical and reference signals using a frequency domain analysis in sliding windows. (**b**,**e**) In each window, the PSD was computed using Welch’s method and the dominant frequency was selected. This analysis was computed using six different window sizes. (**c**,**f**) An example for one subject of the HR and RR values extracted in all the window sizes is also reported.

After the signal segmentation and the PSD assessment, in each window, the HR and RR values expressed in Hz were estimated by detecting the maximum frequency peak within the cardiac and respiratory frequency band, respectively. After multiplying the dominant frequency by a factor of 60, we computed the HR and RR values expressed in bpm and BrPM, respectively. An example is shown in Figure 5. This approach allowed us to obtain a trend of HR and RR values over time with an update time of 1 s for each window size.

To assess the performance of the HR and RR extraction from SCG and GCG signals, Bland–Altman analysis was performed. Bland–Altman analysis is one of the most popular methods applied to investigate the agreement between the same measurement extracted with a new measurement technique and an established one [37]. Specifically, it was used to obtain the mean of difference (MOD) and the limit of agreement (LOA) values that are typically reported in other studies and extremely useful when comparing our results with the relevant scientific literature.

**Figure 6:**
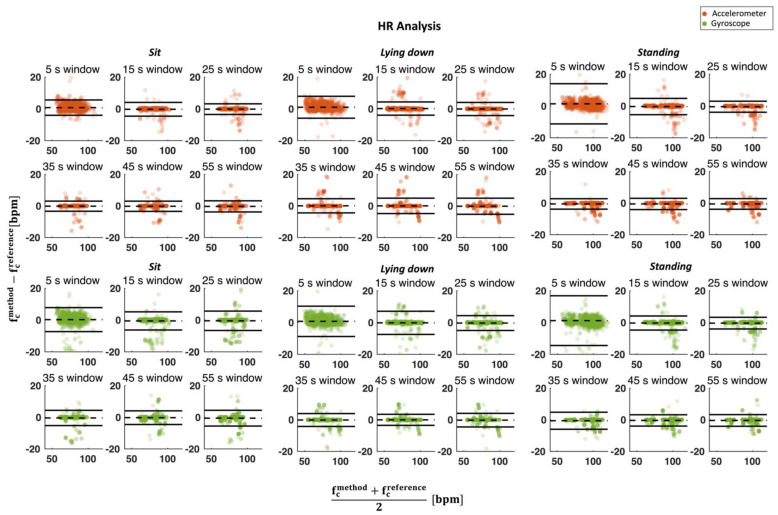
Bland–Altman plot for HR estimation considering all postures from both a (in orange) and ω (in green) signals against reference. The dotted line in the figure represents the MOD, while the solid lines represent the LOA values. In each graph, the number of points is equal to the number of subjects multiplied by 120 (i.e., the duration of the test) minus the length of the window. For example, for the 5 s window, there will be 1256 points for a and 1256 points for ω.

**Figure 7:**
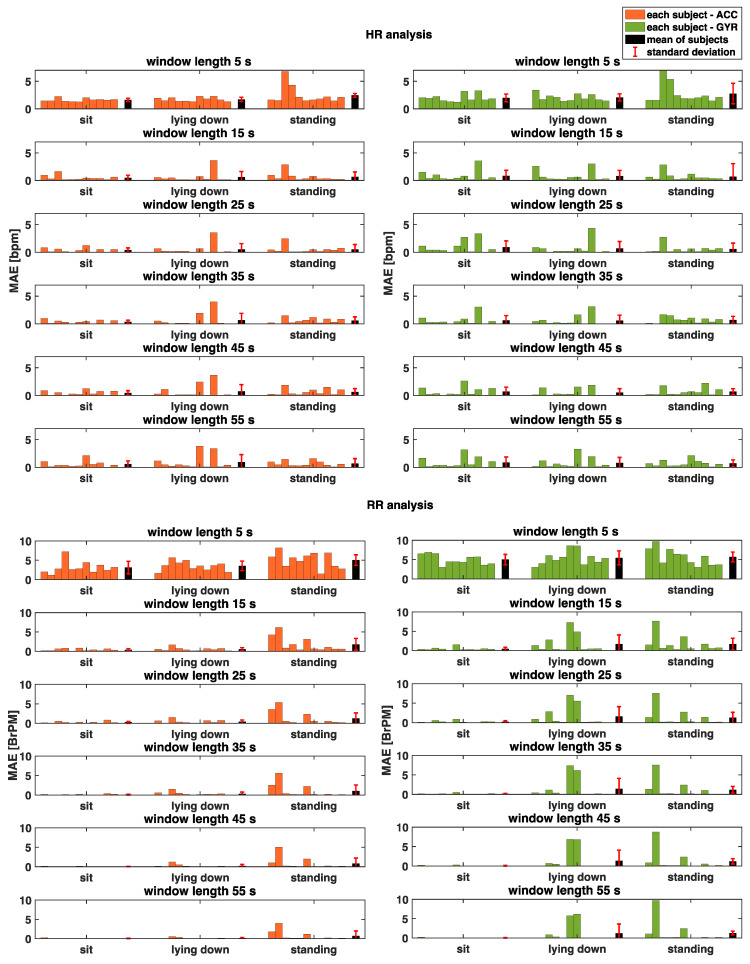
Bar graph of the mean absolute error (MAE) values calculated for both HR analysis (in the upper graph) and RR analysis (in the bottom graph) and for the ACC (in orange) and the GYR (in green). Moreover, these values are reported for each subject tacking on the different postures (i.e., sitting, lying down, and standing) and for all the window lengths. For each analyzed combination, we included the mean MAE value (in black) considering all subjects and the relative standard deviations (reported as an error bar in red).

This analysis was carried out by considering the different at-rest postures separately to assess the influence of posture on the results. Additionally, the different window sizes were compared (see. Figure 6). Moreover, for a better comparison of performance between windows, the mean absolute error (MAE) between the HR and RR values extracted from the mechanical signals and the reference for each window was calculated (see Figure 7).

## 3. Results

### 3.1. Cardiac Activity

Cardiac activity analysis involves the computation of HR values extracted with the frequency domain analysis performed with sliding windows during both quiet breathing and apnea phases. Figure 6 shows the Bland–Altman plot in sitting, lying down, and standing postures, respectively. In Bland–Altman plots, the central dashed line shows the MOD between values estimated with the proposed method and from ECG, while the upper and lower horizontal lines indicate the 95% confidence intervals (LOA values).

After looking at the Bland–Altman plot, the worst results are obtained when a 5 s window is used considering both SCG and GCG. The LOA values are always much larger than in all other windows in the standing posture (12.5 bpm and 12.8 bpm compared to 3.5 bpm and 3.7 bpm obtained with a 55 s window size for SCG and GCG, respectively). Furthermore, the results appear to be roughly comparable using the other five window sizes (i.e., 15 s, 25 s, 35 s, 45 s, and 55 s).

MAE values allow a clear comparison between SCG (in orange) and GCG (in green) to be performed, considering the different window sizes, as shown in Figure 7. The SCG signal gives comparable results to the GCG signal, reaching a difference of 0.53 bpm from the SCG in the seated posture at most. The trend in MAE values, as a function of window size, is also noteworthy. In fact, one notices a sudden descent of MAE values in the transition from 5 s to 15 s in all postures of the subject. From 15 s onwards, on the other hand, the MAE value remains approximately constant up to the 35 s window and then increases slightly, deviating by a maximum of 0.34 bpm considering the windows from 15 s to 55 s.

### 3.2. Respiratory Activity

**Figure 8:**
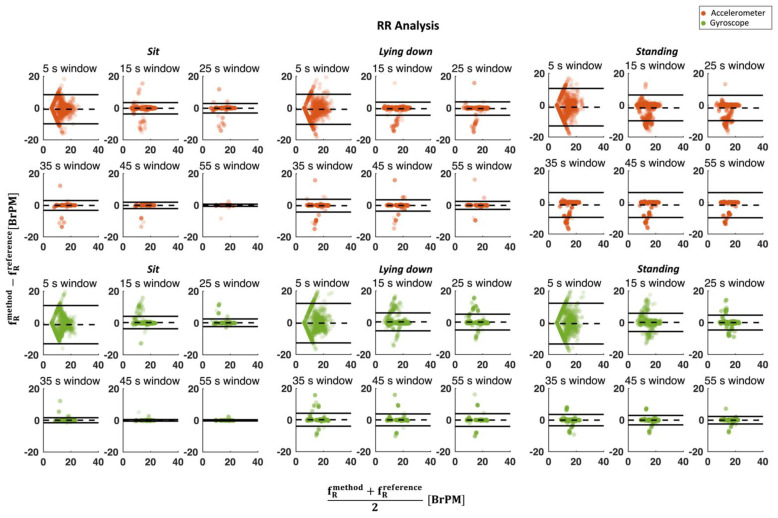
Bland–Altman plot for RR estimation considering all postures from both a (in orange) and ω (in green) signals against reference. The dashed line in the figure represents the MOD while the solid lines represent the LOA values. In each graph, the number of points is equal to the number of subjects multiplied by 120 (i.e., the duration of the test) minus the length of the window.

As for the cardiac analysis, the worst results in the RR estimation are obtained when a 5 s window is used considering both SCG and GCG, reaching LOA values of 13.8 BrPM and 12.9 BrPM during the standing posture, compared to 4.7 BrPM and 4.4 BrPM using a 55 s window size, respectively (see Figure 8). Furthermore, there is an ever-decreasing trend in the results as the window size increases.

MAE values (see Figure 7) show that the GCG signal gives results slightly better than the SCG signal. This result is more evident during the standing posture where the MAE values calculated by GCG are always below those calculated by SCG by at least 1.1 BrPM. Regarding the trend in MAE values, as a function of window size, a decreasing trend can be observed.

## 4. Discussion and Conclusions

The aim of this paper is twofold: (i) to provide a comparison in terms of performance between the ACC and the GYR in the simultaneous extraction of HR and RR values; (ii) to investigate how the window lengths and subjects’ posture influence the extraction of HR and RR values from ACC and GYR signals.

Tests were carried out on eleven subjects, assuming different at-rest postures to mimic common daily-life conditions. The extraction of HR and RR values was performed by computing a frequency domain analysis in sling windows. In an effort to understand if and how the portion of the signal used for the analysis influences the performances of the algorithm, we investigated six different sizes. Although the literature has reported a few studies which performed frequency domain analysis in sliding windows, a comparison between the performances using different sizes has yet to be proposed. This analysis allows us to find the best trade-off between the window length that is related to the ability to track the HR and RR values over time, and the performances in cardiac and respiratory frequency extraction.

In the extraction of the HR, a comparison between the window sizes shows that there is a sudden decreasing trend in MAE values when windows from 5 s to 25 s are used for both ACC and GYR output signals. Afterwards, this trend does not undergo major changes from windows 25 s to 55 s in which always remains below 1 bpm and fulfils the requirement given in ANSI/AAMI EC13: 2002 [38] for both the SCG and the GCG. 

Regarding a 25 s window length, the MOD and LOA values of the data are fairly better than those obtained in [20] where combined ACC and GYR measurements are performed on lying-down subjects. Although the HR extraction method is not exactly the same (MOD ± LOA values = 0.30 ± 5.36 bpm for the ACC and GYR combination reported in [20] against MOD ± LOA values = −0.07 ± 4.12 bpm for the ACC and MOD ± LOA values = −0.32 ± 4.67 bpm for the GYR considering our study), in both cases, lying-down posture was considered.

Furthermore, in [23], authors proposed a dedicated algorithm to estimate heart rate and respiratory rate from an accelerometer by using the CEBS database of PhysioNet and additional experiments for the respiratory rate extraction. The results (in terms of MOD and LOA values) reported in this study with supine subjects are slightly better than those reported in our study regarding the supine position and a window of 25 s.

Regarding RR, the trend is always downward with a steeper slope in the step from 5 s to 15 s and less in subsequent steps, settling the MAE values always below 3 BrPM. Furthermore, the analysis showed that there are no major differences between the ACC and the GYR in the HR extraction, while slightly better results are obtained when the GYR is used for RR extraction compared to the ACC, especially during the standing posture.

Overall, the errors obtained in HR and RR extraction are comparable in terms of MOD and LOA values to studies that focus on only one of the two parameters. For example, [22] proposes the extraction of HR values by using a frequency domain approach from the combination of ACC and GYR output signals with the subject in static positions (i.e., sitting and standing) and an IMU sensor attached to clothes at the chest level. In the standing posture, an MOD of −2.02 with 95% LOA in −21.45 to 17.41 (using the *z* axis of the ACC) is shown, as well as an MOD of −0.07 with 95% LOA in −6.14 to 6.00 (using a combination of the ACC and the GYR). The mean errors during the sitting posture were −8.30 with 95% LOA in −25.35 to 8.75 (using the *z* axis of the ACC) and −3.82 with 95% LOA in −18.70 to 11.07 (using a combination of the ACC and the GYR). These are quite a bit higher than those presented in our study by considering the same postures (both sitting and standing postures) and all the lengths of the windows. In addition, [34] proposes a system embedding two IMU sensors only for RR extraction. However, the use of multiple sensors increases the complexity of both the algorithm and the system. Finally, the achieved results are even comparable to those obtained with other much more cumbersome techniques that can measure both cardiac and respiratory activity [36].

Although there are a few studies comparing the ACC and the GYR, in terms of waveforms, spectra, amplitude intervals, cardiac cycle length and area, and the extraction of HRV indices [13,39], there are few investigations on how the two techniques (i.e., the ACC and the GYR) simultaneously extract HR and RR values considering different postures. Moreover, when frequency domain analysis is performed on the whole signal, only one average HR or RR value is obtained. In order to obtain information regarding the variation in the two vital parameters over time, a segmentation of the signal with moving windows is necessary. By using 1 s as a sliding step, as the size of the window increases, and thus the size of the signal on which the PSD is measured, the ability of the system to follow the changes in HR and RR values over time decreases, especially when these changes are rapid. Hence, the data in two adjacent windows and the HR and RR values are highly correlated, especially when larger windows are used. This phenomenon can be seen from the graphs shown on the right of Figure 5. In fact, it is evident that more pronounced oscillations between the i-th window and the i + 1-th window (represented in the figure by two adjacent dots) occur when smaller lengths of windows are used. Conversely, when higher lengths of windows are used, the oscillations decrease. For these reasons, significant efforts have been devoted to providing guidelines (in terms of window size and user posture) for the simultaneous and reliable estimation of HR and RR values through ACC and GYR output signals. This will push the adoption of the IMU-based wearable systems during daily life activities using a low-cost, minimally invasive device.

A major limitation of this study is the sample size used for analysis and the brevity of the tests conducted, which does not allow an excellent generalization of results. In addition, the HR and RR values were calculated only with the subject at rest. This condition is optimal to avoid motion artifacts affecting the signal; however, a possible future development could be to evaluate the goodness of the developed algorithm for HR and RR estimation, also with moving subjects, thus extending the applicability of the proposed technique for a wider range of applications and scenarios. Moreover, as can be seen in Figure 7, there is variability between subjects, especially in particular cases such as in the case of subject 2 during the standing test when respiratory activity is extracted. These changes can be attributed to typical inter-subject variations, including, gender, age, sensor adherence to the chest, and postural positions, as the literature on these types of signals suggests [13,25].

In conclusion, this study demonstrates that both the ACC and the GYR embedded in a single IMU sensor can estimate HR and RR values. Better results were obtained using the ACC output when compared to the GYR. Moreover, we provide guidelines for signal analysis when a frequency domain approach is used, highlighting the system potentiality of real-time cardiorespiratory monitoring. However, this analysis can provide different results considering trials on a sample, showing both a lower or higher variability of HR and RR values.

## Data Availability

The data presented in this study are available on request from the corresponding author. The data are not publicly available due to privacy reasons.
